# Steady-State Visual-Evoked Potentials as a Biomarker for Concussion: A Pilot Study

**DOI:** 10.3389/fnins.2020.00171

**Published:** 2020-03-10

**Authors:** Daryl H. C. Fong, Adrian Cohen, Philip Boughton, Paul Raftos, Joseph E. Herrera, Neil G. Simon, David Putrino

**Affiliations:** ^1^School of Aerospace, Mechanical and Mechatronic Engineering, Faculty of Engineering and Information Technologies, The University of Sydney, Sydney, NSW, Australia; ^2^Save Sight Institute, Sydney Medical School, The University of Sydney, Sydney, NSW, Australia; ^3^Randwick District Rugby Union Football Club, Sydney, NSW, Australia; ^4^Department of Rehabilitation and Human Performance, Icahn School of Medicine at Mount Sinai, New York City, NY, United States; ^5^Northern Clinical School, The University of Sydney, Sydney, NSW, Australia

**Keywords:** concussion, sport, electroencephalography, SSVEP, football

## Abstract

A variety of assessment tools are currently available to help clinicians assess Sports Related Concussion (SRC). Currently, the most widely available tools are neither objective nor portable, and are therefore not ideal for assessment at the site and time of a suspected injury. A portable system was developed to deliver a measurement of the steady-state visual-evoked potential (SSVEP). This system involved a smartphone housed in a Google Cardboard frame, which delivered a 15-Hz flicker visual stimulus while an electroencephalography (EEG) headset recorded EEG signals. Sixty-five rugby union players were tested during their regular season and were stratified into healthy, concussed, and recovered groups based on clinical examination. Their SSVEP response was quantified into a signal-to-noise ratio (SNR). The SNRs of players in each study group were summarized. Additionally, the SNRs of individual players who had baseline, post-injury, and post-recovery readings were analyzed. Sixty-five participants completed a baseline evaluation to measure their SSVEP. Twelve of these participants sustained a medically diagnosed concussion and completed SSVEP re-testing within 72 h. Eight concussed players received follow-up SSVEP testing after recovery. Concussed participants had a lower SNR [2.20 (2.04–2.38)] when compared to their baseline [4.54 (3.79–5.10)]. When clinically recovered, participant SNR was not significantly different to their baseline [4.82 (4.13–5.18)]. The baseline SNRs of the players who experienced a concussion during the season were not different to those of players who did not experience a concussion [4.80 (4.07–5.68)]. This is the first study to identify differences in SSVEP responses in male amateur rugby union players with and without concussion. It is also the first SSVEP demonstration for concussion evaluation at point-of-care. SSVEPs are significantly attenuated in the presence of concussion in these male athletes. Individuals returned to their baseline SSVEP following clinical recovery from the concussive injury. The use of SSVEPs has the potential to be a supplemental aid for the assessment and management of concussion.

## Introduction

Sports-related concussion (SRC) is defined by the Concussion in Sport Group as “*immediate and transient symptoms immediately following a mild traumatic brain injury occasioned during sport*” (McCrory et al., 2017). SRC is caused by a direct force delivered to the head or anywhere else on the body which results in impulsive force being transmitted to the brain (McCrory et al., 2017). In some cases, signs and symptoms evolve over a number of minutes to hours, and in most cases resolve spontaneously by 7–10 days (Graham et al., 2014) provided the individual is not exposed to further impacts.

Concussion is a common injury in contact sports, with an incidence rate of approximately 1 per 1,000 athletic exposures in the NFL (Kilcoyne et al., 2014), and up to 4 concussions per 1,000 player-match-hours in elite rugby union (Gardner et al., 2014; Fuller et al., 2015). The increasing awareness of concussion has motivated sports governing bodies to implement protocols to improve player safety (Partridge and Hall, 2015). The most pressing issues in relation to concussion involve accurate and timely diagnosis, and safe return-to-play criteria (Graham et al., 2014; Levin and Diaz-Arrastia, 2015; Murray et al., 2015). Athletes may minimize or deny symptoms to avoid a concussion diagnosis and accelerate their return to play, potentially increasing the risk of Second Impact Syndrome (Bey and Ostick, 2009). Over long periods, repeated and inappropriately managed concussion may lead to chronic neurological impairment and the possibility of chronic traumatic encephalopathy (CTE; Arciniegas, 2011; Stern et al., 2011; Weinstein et al., 2013).

The most commonly used concussion assessment tool is the multi-modal Sport Concussion Assessment Tool (SCAT; Echemendia et al., 2017b) which incorporates cognitive assessment, symptom reporting, physical examination, coordination, and balance testing (Giza et al., 2013). Other computerized neurocognitive tests (CNT) require active participation from the participant and thus are influenced by participant motivation and effort, prone to ceiling effects (Echemendia et al., 2017a) and suffer poor reliability (Ragan et al., 2009; Echemendia et al., 2017a). Moreover, these assessment tools are vulnerable to manipulation by athletes despite checks built into tests like the Immediate Post-Concussion Assessment Tool (ImPACT; Alsalaheen et al., 2016). Symptom reporting is also subjective and unreliable.

Due to the limitations of existing tests and the growing realization of the significance of the condition, there is a critical need for an objective biomarker of SRC that can be rapidly applied to athletes, ideally at the point of occurrence. The steady-state visual-evoked potential (SSVEP) is an objective, quantifiable fluctuation of electrical activity that occurs in the brain in response to a specific set of visual stimuli, and is measurable using EEG technology (Phurailatpam, 2014; Kothari et al., 2016). Since the discovery of the SSVEP in the 1950s, it has become an important tool for understanding the relationships between physical stimuli, brain activity, and human cognition (Galloway, 1990; Handy, 2005; Luck and Kappenman, 2011). There is emerging evidence that VEPs are chronically impaired following a concussion (Freed and Hellerstein, 1997; Boutin et al., 2008), with studies showing signal abnormalities or significant attenuation in study participants following the diagnosis of a concussion (Freed and Hellerstein, 1997; Moore et al., 2014; Yadav and Ciuffreda, 2015; Poltavski et al., 2017). Thus, VEPs may be a useful objective biomarker of concussion.

There are several advantages of using SSVEPs compared with conventional VEPs such as: (1) lack of synchronicity between EEG recorder and visual stimulus (simplifying equipment requirements), (2) relative resistance to noise artifacts, and (3) improved resilience to variable contact impedance (Peterson et al., 2014; Dreyer and Herrmann, 2015; Norcia et al., 2015). These advantages make SSVEPs a favorable brain signal to study in non-clinical environments such as on the sideline of sports grounds and in a doctor’s office. The goal of this study was to investigate potential differences in SSVEPs that were recorded from athletes who were assessed by an experienced sports doctor as healthy, concussed, or recovered from a recent concussion.

## Materials and Methods

A prospective cohort observational study was undertaken over a season of rugby union practice and match activities. The South Eastern Sydney Local Health District human research ethics committee approved all procedures in the study [South East Sydney Local Health District HREC ref no: 17/039 (HREC/17/POWH/91)], and all players provided informed written consent prior to participation.

### Participants

**FIGURE 1:**
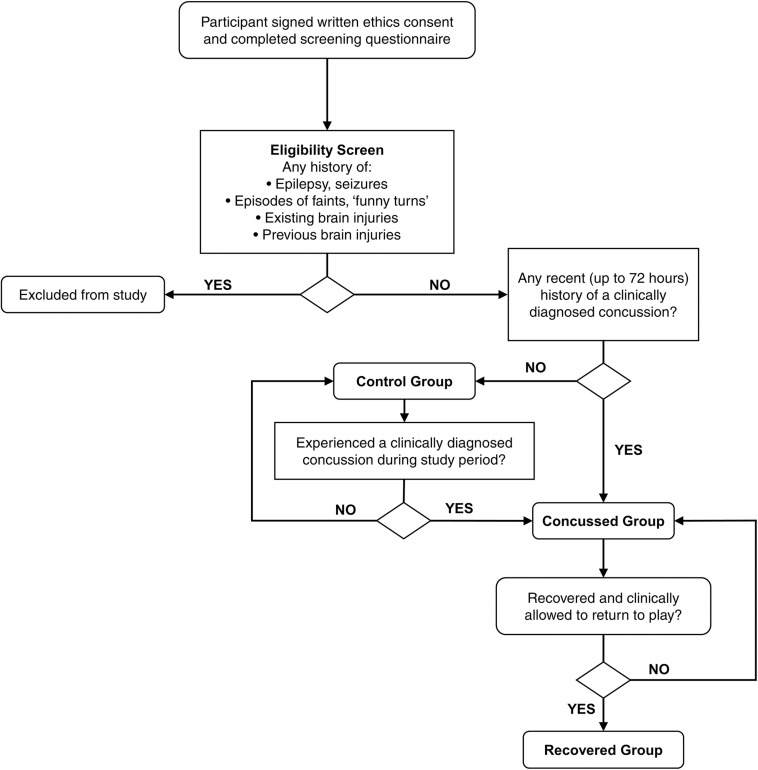
Flowchart of screening for participant eligibility and allocation throughout the study duration of amateur rugby union players.

Healthy members of a male amateur community rugby union team competing in a premier grade club tournament were recruited to participate in the study. Player screening was undertaken to identify any relevant exclusion criteria, and for history of recent concussion (Figure 1). Exclusion criteria included a diagnosis or symptoms of epilepsy, existing and/or previous brain injury, or legal blindness. Testing was performed prior to practice sessions during the regular competition season over 18 weeks in a quiet setting.

### SSVEP Testing Protocol

**FIGURE 2:**
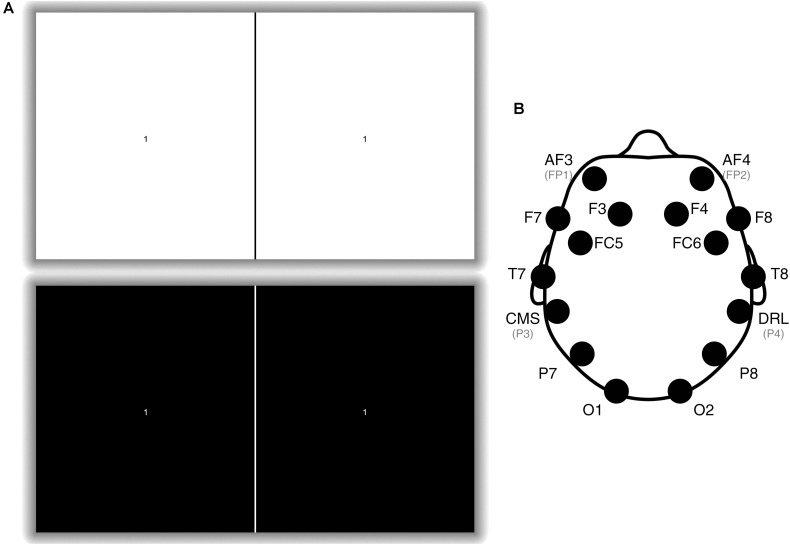
**(A)** An example of the visual stimulus. The stimulus alternated between the top and bottom picture at a rate of 15 times per second. There is a fiducial line in the middle used to align the screen with the Google Cardboard headset. The number at the center of each square changed at 5 s intervals and participants were instructed to focus on the number for a total of 30 s. NB, The shadow does not exist on the actual stimulus but is utilized here to make the visual stimulus clearer to view. **(B)** Emotiv EPOC+ electrode positions (Liu et al., 2012). Only electrodes P3 (CMS), P4 (DRL), O1, and O2 were utilized: P3 and P4 were utilized as a common-mode subtraction/driven-right-leg reference and ground, and O1 and O2 were the analyzed electrodes.

The visual stimulus used for this study (Figure 2A) was displayed to participants using a Sony Xperia Z1 smartphone playing a MP4 video file which lasts for 30 s. The smartphone was placed in a Google Cardboard frame and the participant held this to their head centered over the bridge of the nose covering both eyes. The Google Cardboard housing for the smartphone provided a consistent eye distance to the visual stimulus on each individual tested as it is non-adjustable. The MP4 video comprised a sequence of black and white screens alternating at a frequency of 15 Hz. This frequency was chosen as it falls within the ideal SSVEP frequency range (between 10 and 20 Hz) (Kuś et al., 2013) while also having a 50% duty cycle on the display (i.e. two frames bright, two frames dark). A number was placed in the middle of the screen (occupying <2% of the screen with a visual angle of 1.5°) to allow participants to focus centrally to maximize participant concentration and field of view covered by the stimulus. This number changed at 5 s intervals to encourage sustained attention. All participants were asked to confirm that they could see the fixation target with both eyes prior to testing.

The EEG recordings were measured with an Emotiv EPOC+, a wireless, 14-channel EEG headset. The electrodes were arranged according to the International 10–20 system (Figure 2B; Handy, 2005; Luck and Kappenman, 2011). The O1 and O2 electrodes were used as the main recording electrodes and the P3 and P4 electrodes were utilized as the reference and common-mode electrodes, respectively (these were non-reconfigurable factory settings). Data were sampled at 128 Hz and wirelessly transferred to a laptop computer via the Emotiv Xavier software (v3.1.21) as a European Data Format (EDF) file.

The captured EDF file data were imported into MATLAB 2015b. The entire 30-s recording was used as the analysis window for calculation of the frequency response. A band-pass Butterworth filter with corner frequencies at 5 and 40 Hz was applied to minimize lower-frequency noise, DC voltage offset and mains power. A Fast Fourier Transformation (FFT) was then applied to generate a power spectrum density (Liu et al., 2012). Channels O1 and O2 were summed to generate a single plot. The magnitude at 15 Hz was divided by the average magnitude between 5 and 40 Hz to establish the magnitude ratio (signal-to-noise ratio or SNR). The SNR was utilized for comparison purposes across the different groups. Each participant underwent the SSVEP assessment protocol twice in succession on each testing occasion; the second reading from the two assessments was selected for comparison as it consistently yielded a clearer SNR.

To ensure an adequate connection between the headset and the participant’s head, the Emotiv TestBench software’s contact quality indicator was checked before the test was undertaken. The headset housing the smartphone was provided to the participant; they were instructed to hold it up to their eyes and stare at the number at the center of the screen. The test was then repeated.

Prior to the competition season, all enrolled players underwent a baseline SSVEP assessment. All retests (whether for test–retest reliability or post-injury) were performed prior to the practice session which occurred 2 days after a competition game.

Following a medically diagnosed concussion, an SSVEP reading was acquired within 2 days of injury to assess for any change. Concussed participants were also reassessed 7–14 days after the event for their “recovered” reading once deemed recovered by the team physician.

### Clinical Protocol for Evaluation of a Concussion

All clinical concussion evaluations were performed by the team physician, a general practitioner with 30 years of clinical experience in assessing sports-related injuries and approved by Rugby Australia for competency in providing immediate care in sport.

After witnessing an impact on the field suggestive of compromising the player, having one reported to him by a team official or player, or at the request of an individual player, the team doctor performed an assessment based on elements of the SCAT (Echemendia et al., 2017b). This included questioning of the player regarding orientation in time, place and person, memory of the events, as well as common symptoms of concussion, e.g. headache, nausea, dizziness and balance problems, blurred vision/visual disturbances, confusion, or a feeling of slowness or fatigue. A physical examination was also conducted including evaluation of the central and peripheral nervous system. Diagnosis of concussion was made based on this assessment in association with background knowledge of the player’s typical demeanor and behavior.

Re-assessment by the same team physician occurred within 48 h and again several times during non-contact practice following Rugby Australia’s Graduated Return to Play (GRTP) guidelines (Rugby Australia, 2018), and again before returning to full-contact practice (minimum 12 days post-injury).

### Statistical Analysis

Statistical analysis was performed utilizing IBM SPSS 24. A Shapiro–Wilk normality test determined the SNR distribution to be normally distributed (baseline *W* = 0.97; *p* = 0.2902, concussed *W* = 0.96; *p* = 0.4154, recovered *W* = 0.90; *p* = 0.5987). Paired two-tailed *t*-tests were performed for players who had all three readings (*t*-tests performed between baseline–concussed, baseline–recovered) and a Bonferroni correction was applied to account for multiple comparisons. Test–retest reliability was estimated using an intra-class correlation coefficient (ICC), with 95% confidence intervals (CIs), to examine agreement between baseline and repeated testing throughout the season. Cohen’s effect size (*d*) was used to calculate practically meaningful differences between baseline, concussed, and recovered. Effect sizes of <0.19, 0.20–0.60, 0.61–1.20, and >1.20 were considered trivial, small, moderate, and large, respectively (Yadav and Ciuffreda, 2015). All summarized data are expressed as medians with 25th to 75th interquartile range. Statistical significance level was set at α = 0.05.

## Results

A total of 65 male players (20.9 ± 2.3 years old) were enrolled in the study. No adverse events were recorded during the study. Through the course of the rugby season, 12 participants sustained a concussion that was diagnosed by the team physician and had a second SSVEP assessment. All 12 concussed participants recovered clinically: eight of these were available for further SSVEP assessment, the other four were lost to follow-up.

**FIGURE 3:**
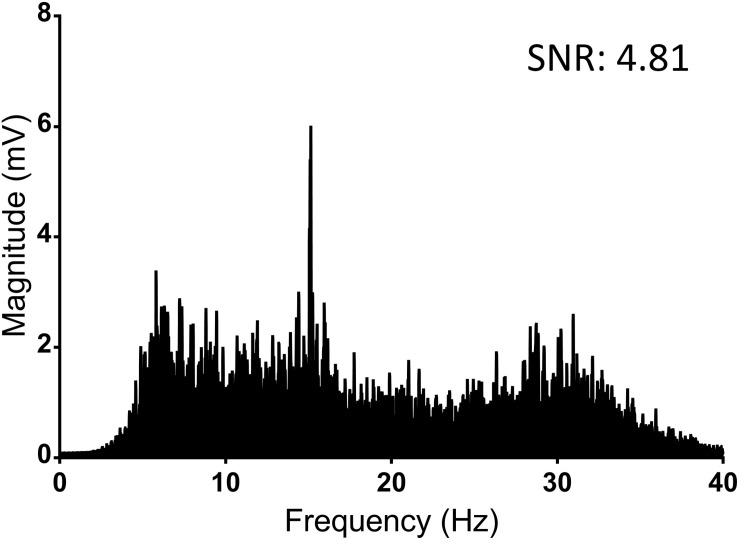
Average Fourier transformation of the frequency spectrum (SSVEP) of all 65 control players (baseline). This includes the retest results of the non-concussed players. SNR, signal to noise ratio.

Overall, the median SNR for all 65 players was 4.80 [interquartile range (IQR): 4.07–5.68]. For the 12 concussed players, the median SNR was 2.00 (IQR: 1.40–2.32). For the eight recovered players who were available for a further SSVEP assessment, the median SNR was 4.82 (IQR: 4.13–5.18) (Table 1 and Figure 3).

**TABLE 1:** SSVEP SNR values of total participants and participants that recorded a concussion.

	SNR	vs. Baseline	vs. Concussed	vs. Recovered
				
Group	Median [IQR]	diff (*p*-value); *d*=	diff (*p*-value); *d*=	diff (*p*-value); *d*=
**All participants (*n* = 65)**				
Baseline	4.80 [4.07–5.68]	–	+2.80 (<0.0001); 4.03	−0.02 (0.0117); 0.40
Concussed	2.00 [1.40–2.32]	−2.80 (<0.0001); 4.03	–	−2.82 (<0.0001); 5.25
Recovered	4.82 [4.13–5.18]	+0.02 (0.0495); 0.17	+2.82 (0.0002); 3.60	–
**Participants with baseline, concussed, and recovered SSVEPs (*n* = 8)**				
Baseline	4.54 [3.79–5.10]	–	−2.25 (0.0001); 4.20	−0.28 (0.0495); 0.17
Concussed	2.20 [2.04–2.38]	−2.34 (0.0001); 4.20	–	−2.72 (0.0002); 3.60
Recovered	4.82 [4.13–5.18]	+0.28 (0.0495); 0.17	−2.72 (0.0002); 3.60	–

*These numbers include retests.*

**FIGURE 4:**
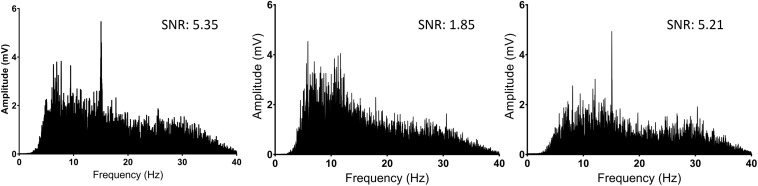
Fourier transformations of the frequency spectrum (SSVEP) comparisons of player JJ2 for baseline **(Left)**, concussed **(Center)**, and when clinically recovered **(Right)**. Note the presence of a peak at 15 Hz on the left and right figures, demonstrating a response to the 15 Hz visual stimulus. SNR for each reading is also noted for each graph. SNR; signal to noise ratio.

Changes were observed in the stimulus response strength (SNR) in the identified concussed participants when compared to their baseline [2.00 (95% CI: 1.83–2.16) vs. 5.01 (4.78–5.24); *p* < 0.0001] (Table 1 and Figure 4). The eight players who were re-evaluated after recovery had an increased SNR compared to their concussed SSVEP (Table 2).

**TABLE 2:** Individual SSVEP response (SNR) for players at baseline, immediately after concussion, and subsequent recovery.

Player	Baseline	SNR concussed	Recovered	BL vs. Conc.	Differences identified Conc. vs. Rec.	Rec. vs. BL
1	4.52	2.33	4.24	2.19	−1.92	−0.28
2	3.32	2.05	3.10	1.27	−1.05	−0.22
3	4.55	2.31	4.68	2.24	−2.37	0.13
4	3.99	2.03	3.77	1.95	−1.74	−0.22
5	5.09	2.09	5.17	3.00	−3.09	0.09
6	5.35	1.85	5.21	3.50	−3.36	−0.14
7	3.72	2.51	3.48	1.21	−0.98	−0.23
8	5.10	2.40	4.96	2.70	−2.55	−0.15
9	5.71	2.62	N/A	3.09	N/A	N/A
10	4.19	2.31	N/A	1.88	N/A	N/A
11	5.21	1.98	N/A	3.23	N/A	N/A
12	5.18	2.15	N/A	3.03	N/A	N/A
Average	4.45	2.20	4.33	2.26 (*p* = 0.0001) (Matched) 2.34 (Unmatched)	−2.13 (*p* = 0.0002) (Matched)	0.13 (*p* = 0.0495) (Matched)

*The baseline SNR was acquired before a concussion occurred; the concussed SNR up to 72 h after a concussion; the recovered SNR after being clinical recovery; BL, baseline; Conc., concussed; Rec., recovered. The calculated *p*-values are from the paired *t-*tests of the first eight players only. Matched averages are calculated only from the first eight players.*

Twenty-two players who were not concussed were retested over the season: the remaining players were lost to follow-up. The ICC between the first and second tests for non-concussed participants was 0.91 (95% CI: 0.79–0.96) (Table 3). Mean time between testing was 31.91 ± 11.22 days for players that did not sustain a concussion.

**TABLE 3:** Test–retest reliability of the SSVEP findings for players who have undergone multiple testing throughout the season.

Group	*N*=	ICC (95% CI)	Mean time
Baseline	22	0.91 (0.79–0.96)	31.91 ± 11.22
Recovered	5	0.96 (0.74–0.99)	17.60 ± 6.23

*Baseline test–retests were performed on players periodically over the season who had not recorded a concussion; recovered test–retests were performed periodically on players who were concussed but had since clinically recovered. Mean time: mean time between testing.*

## Discussion

This is the first study to identify differences in SSVEP response in healthy active male amateur rugby union players when concussed, and the first study to demonstrate the use of SSVEP at point-of-care. This makes it an important step to a potential objective biomarker of concussion. In addition, the fact that an SSVEP is logistically easier to acquire than a VEP makes the goal of a simple sideline assessment more realistic. The study also demonstrated a return to pre-concussion SSVEP values following clinical recovery. High test–retest reliability in non-concussed participants highlights the stability of the measurement, even when the repeated testing was conducted several weeks apart.

Previous studies have noted that conventional VEPs are altered by the presence of concussion (Freed and Hellerstein, 1997; Moore et al., 2014; Yadav and Ciuffreda, 2015). In these studies, the main findings demonstrate attenuated or delayed VEP responses. This is consistent with our findings that the SSVEP was attenuated in the presence of concussion. Furthermore, our study builds on the existing literature by showing differences in SSVEP in baseline, concussed, and recovered individuals as opposed to control and injured cohorts.

As previously noted, diagnosis of concussion that relies on subjective criteria is far from ideal. Radiological modalities such as magnetic resonance imaging (MRI) and computed tomography (CT) provide information about macroscopic structural injuries (Slobounov and Sebastianelli, 2014). Concussion is not a macroscopic structural injury; therefore, these modalities are primarily used to rule out injuries such as hemorrhage (Graham et al., 2014). As neurophysiological biomarkers, VEP (and SSVEP) assesses function rather than structural integrity (Drislane, 2007). The changes in the SSVEP response found in our concussed participants likely represent a disruption of neuronal function. Whether this is due to primary (e.g. damaged white matter) or secondary phenomena (such as inflammatory response) is yet to be established, and further studies are recommended to explore this.

Several limitations to this study were noted. The background EEG noise was variable even among the same individuals tested again immediately after their first test. Possible reasons were hypothesized to be the cause: (1) poor impedance control (as the system did not feedback the actual impedance values) and (2) variable visual focus during tests due to fatigue or distractions. Although the Epoc+ is sufficiently accurate to capture SSVEPs (Liu et al., 2012), correlation with results from other EEG equipment may provide a deeper insight into whether these variances are naturally occurring, or a shortcoming of the current equipment. In most participants the second SSVEP SNR was larger than the first. This may be due to familiarization with the process and less blinking during repeat stimulus presentation. Further studies are needed to determine if there is a familiarization effect to the SSVEP protocol.

Further research is required to better understand mechanisms underlying our finding that SSVEPs attenuate in concussion, but this study provides pilot evidence for an objective measure of concussion that is able to be delivered expediently on the sideline. This has the potential to provide significant benefit to medical professionals and coaches, as well as athletes and their families. As this study only assessed 65 players, 12 of whom suffered a concussion, studies with larger sample sizes will assist to evaluate the robustness of the SSVEP in the practical assessment of concussion, particularly its sensitivity and specificity. Additionally, experimentation with different parameters such as varying stimuli time and frequencies on both concussed and non-concussed individuals may be explored in the future to further validate the use SSVEPs in this application.

## Conclusion

In conclusion, SSVEPs offer new potential in the assessment of concussion, by non-invasively and objectively measuring brain function. This study determined that SSVEPs are significantly attenuated in the presence of concussion in male athletes. It also shows that individuals return to their baseline SSVEP following recovery from the concussive injury. The use of SSVEPs has the potential to be a supplemental aid for the assessment of concussion. Further studies with larger cohorts, as well as comparing SSVEPs to other methods of concussion diagnosis will be necessary to fully understand the potential of this technique.

## Data Availability Statement

The datasets generated for this study are available on request to the corresponding author.

## Ethics Statement

The studies involving human participants were reviewed and approved by the South East Sydney Local Health District Human Research Ethics Committee. The patients/participants provided their written informed consent to participate in this study.

## Author Contributions

DF, AC, and PB contributed to the conception and design of the study. PR performed the clinical assessments. DF and PR collected the data. DF performed the statistical analysis and wrote the first draft of the manuscript. All authors contributed to the manuscript revision, and read and approved the submitted version of the manuscript.

## Conflict of Interest

DF was employed by Cryptych Pty Ltd. and received institutional financial and in-kind support from HeadsafeIP for the work described. AC is a director of and owns shares in HeadsafeIP. PB, PR, and NS declare no financial or other relationships associated with the research. No financial or other relationship exists between the Randwick District Rugby Union Football Club and the work described. DP and JH declare institutional support received from HeadsafeIP.
