# Corrigendum: Steady-State Visual-Evoked Potentials as a Biomarker for Concussion: A Pilot Study

**DOI:** 10.3389/fnins.2020.00866

**Published:** 2020-10-05

**Authors:** Daryl H. C. Fong, Adrian Cohen, Philip Boughton, Paul Raftos, Joseph E. Herrera, Neil G. Simon, David Putrino

**Affiliations:** ^1^School of Aerospace, Mechanical and Mechatronic Engineering, Faculty of Engineering and Information Technologies, The University of Sydney, Sydney, NSW, Australia; ^2^Save Sight Institute, Sydney Medical School, The University of Sydney, Sydney, NSW, Australia; ^3^Randwick District Rugby Union Football Club, Sydney, NSW, Australia; ^4^Department of Rehabilitation and Human Performance, Icahn School of Medicine at Mount Sinai, New York City, NY, United States; ^5^Northern Clinical School, The University of Sydney, Sydney, NSW, Australia

**Keywords:** concussion, sport, electroencephalography, SSVEP, football

In the original article, we neglected to include that one of the authors, AC, has filed a patent for technology in this general area and that a company HeadsafeIP provided indirect funding to DF, DP, and JH.

The authors apologize for this error and state that this does not change the scientific conclusions of the article in any way. The original article has been updated.

